# Risk Factors for COVID-19 Mortality in Malaysia

**DOI:** 10.21315/mjms2022.29.6.12

**Published:** 2022-12-22

**Authors:** Tengku Muhammad Hanis, Wan Nor Arifin, Kamarul Imran Musa, Wan Shakira Rodzlan Hasani, Che Muhammad Nur Hidayat Che Nawi, Shahnon Anuar Shahrani, Xin Wee Chen, Mohd Azmi Suliman, Erwan Ershad Ahmad Khan, Wira Alfatah Ab Aziz, Mohamad Zarudin Mat Said

**Affiliations:** 1 Department of Community Medicine, School of Medical Sciences, Universiti Sains Malaysia, Kelantan, Malaysia; 2 Biostatistics and Research Methodology Unit, School of Medical Sciences, Universiti Sains Malaysia, Kelantan, Malaysia; 3 Institute for Public Health, National Institutes of Health, Ministry of Health Malaysia, Selangor, Malaysia; 4 KMI Kuala Terengganu Medical Center, Terengganu, Malaysia; 5 Department of Public Health Medicine, Faculty of Medicine, Sungai Buloh Campus, Universiti Teknologi MARA, Selangor, Malaysia; 6 Pejabat Kesihatan Daerah Kota Bharu, Ministry of Health Malaysia, Kelantan, Malaysia; 7 Pejabat Kesihatan Daerah Batang Padang, Ministry of Health Malaysia, Perak, Malaysia

**Keywords:** COVID-19, Malaysia, mortality, risk factor, vaccination

## Abstract

**Background:**

Understanding the risks of COVID-19 mortality helps in the planning and prevention of the disease. This study aimed to determine the risk factors for COVID-19 mortality in Malaysia.

**Methods:**

Secondary online data provided by the Ministry of Health, Malaysia and Malaysia’s national COVID-19 immunisation programme were used: i) COVID-19 deaths data; ii) vaccination coverage data and iii) population estimate data. Quasi-Poisson regression was performed to determine the risk factors for COVID-19 mortality.

**Results:**

Four risk factors were identified: i) vaccination status (partial versus unvaccinated, incidence rate ratio [IRR]: 0.59; 95% CI: 0.54, 0.64; complete versus unvaccinated, IRR: 0.50; 95% CI: 0.45, 0.56; booster versus unvaccinated, IRR: 0.13; 95% CI: 0.05, 0.26); ii) age group (19 years old–59 years old versus above 60 years old, IRR: 0.90; 95% CI: 0.84, 0.97; 13 years old–18 years old versus above 60 years old, IRR: 0.09; 95% CI: 0.04, 0.19; 6 years old–12 years old versus above 60 years old, IRR: 0.09; 95% CI: 0.03, 0.22; below 5 years old versus above 60 years old, IRR: 0.11; 95% CI: 0.04, 0.23); iii) gender (male versus female, IRR: 1.23; 95% CI: 1.14, 1.32) and iv) comorbidity (yes versus no, IRR: 2.13; 95% CI: 1.96, 2.32).

**Conclusion:**

This study highlighted the risk factors for COVID-19 mortality and the benefit of COVID-19 vaccination, especially of booster vaccination, in reducing the risk of COVID-19 mortality in Malaysia.

## Introduction

In December 2019, the first pneumonia cases of unknown origin—later identified as Coronavirus Disease 2019 (COVID-19)—were detected in Wuhan, China. Since then, the disease began to spread rapidly across mainland China and all over the world. On March 11, 2020, the WHO declared COVID-19 as a pandemic (1). As of 22 February 2022, COVID-19 had infected over 423 million (423,437,674) people worldwide with more than 5.8 million (5,878,328) deaths (2). Malaysia reported 3,273,958 cumulative confirmed cases on the same day with 32,433 deaths (3).

With the emergence of the COVID-19 pandemic in 2020, the whole world faced changes of local and global proportions in terms of economic impact and social adaptations required to mitigate the spread of the virus. Different countries formulated different containment and mitigation strategies to combat the disease, including lockdowns and curfews, while anxiously waiting for the arrival of a definitive tool to fight it. Non-pharmaceutical measures, such as social distancing, self-isolation, closure of non-essential services and border control, were successful in limiting infections in the community up to early 2020 The impact on the healthcare infrastructure in ASEAN countries was also fairly mild in 2020 compared to that in 2021 (4).

However, the emergence of variants of concern, specifically the delta variant, resulted in a rapid increase in the number of cases. By the second half of 2021, countries in the South-East Asian region were heavily burdened with the number of new infections. Indonesia peaked in late July 2021 with more than 50,000 new cases per day, while by August 2021, Malaysia peaked with almost 25,000 new cases per day (5). The presence of the more infectious delta variant (6) and the low vaccination coverage at the time are two of the main factors in the high number of cases.

Vaccine development was accelerated to provide immunity to the virus and prevent its transmission (7). As of mid-2021, 3 billion doses of the COVID-19 vaccine had been administered worldwide, with more than 40 million doses being administered worldwide every day (8). After the global vaccination, it was found that the risks of COVID-19 infection, hospitalisation and death were significantly reduced in vaccinated individuals (9). Malaysia started its vaccination programme against COVID-19 in early March 2021. Frontliners, including health workers, were prioritised in the first phase of vaccination, followed by high-risk groups in the second phase and the general adult population in the third phase. As of 1 March 2022, 78.8% and 44.6% of the Malaysian population had received at least two doses and the booster dose of the COVID-19 vaccine, respectively (10).

With COVID-19 rapidly impacting mortality worldwide, it is crucial to understand and assess the mortality risk of COVID-19 infection through timely data analysis. Conventional approaches that have been used in research so far, such as extracting data from electronic health records, led to late reports, which resulted in delayed actions. For example, in the case of the National Cancer Registry in Sabah, Malaysia, the time taken from initial diagnosis to recording and from recording to reporting may vary from 6 months to 4 years (11). This lag is due to decentralised data management and sharing within and between ministries and other organisations. Since July 2021, Ministry of Health (MOH) Malaysia has enabled transparent COVID-19 data sharing by creating publicly accessible GitHub repositories. This move is expected to allow timely data analysis and decision making to better understand the COVID-19 situation in Malaysia. Hence, the objective of this study was to determine risk factors for COVID-19 mortality in Malaysia on the basis of publicly available data.

## Methods

### COVID-19 Data Sources

Three publicly available data were used in this study. All the data were accessed and downloaded on 18 February 2022. The data are listed as follows:

- COVID-19 deaths line list data available from the official GitHub account of the Ministry of Health, Malaysia (https://github.com/MoH-Malaysia/covid19-public/tree/main/epidemic/linelist)
- Vaccination coverage data available from the official GitHub account of Malaysia’s national COVID-19 immunisation programme (PICK) (https://github.com/CITF-Malaysia/citf-public/tree/main/vaccination)
- Malaysian population data available from the official GitHub account of the Ministry of Health Malaysia (https://github.com/MoH-Malaysia/covid19-public)

### Variables

The COVID-19 deaths line list data included the following variables: date of death, date of publicly announced death, date that the sample tested positive, dates of the first, second and booster doses taken, type of vaccine taken at each dose, state of residence, age, gender, brought-in-dead status, Malaysian nationality and comorbidity status. The data covered COVID-19 deaths from 15 February 2020, until the date of data access.

The vaccination coverage data included the following variables: date; state; daily number of partial vaccinations (first dose of double-dose vaccines); daily number of complete vaccinations (second dose of double-dose vaccine and first dose of first-dose vaccine); daily number of booster vaccinations; daily number of partial, complete and booster vaccinations for adults (above 18 years old) and children (18 years old and below); total daily vaccinations; cumulative number of partial, complete and booster vaccinations; cumulative number of partial, complete and booster vaccinations for adults (above 18 years old) and children (18 years old and below); number of recipients of Pfizer, Sinovac, AstraZeneca and Sinopharm vaccines as the first dose, second dose and booster dose; number of recipients of CanSino vaccine as the first dose and booster dose; and pending status. Pending status reflected the pending delivered doses due to backdated manual uploads. The coverage data included all vaccination records from 24 February 2021, until the date of data access.

The Malaysian population data included the following variables: state, total population estimate and population estimate for each age group: 5 years old and below, 5 years old–12 years old, 18 years old and above, 60 years old and above. The population data were based on the latest 2021 estimates.

The vaccination coverage data and Malaysian population data were then used to calculate the daily percentage of second dose vaccination coverage over time. The following formula was used:


$$\text{Daily second dose coverage}=\frac{\text{Daily cumulative number of second dose}}{\text{Total Malaysian population}}\times 100$$

This variable was then integrated into the COVID-19 deaths data.

In addition, the vaccination status variable was generated. The status was categorised as follows:

- Unvaccinated: Death cases who did not receive any vaccination.
- Partial vaccination: Death cases who received the first dose of double-dose vaccines, received the second dose of double-dose vaccines but died within 2 weeks after the second dose of vaccination or received a dose of single-dose vaccines but died within 2 weeks after the first dose of vaccination.
- Complete vaccination: Those who died after 2 weeks of receiving the second dose of double-dose vaccines or a dose of single-dose vaccines or died within 2 weeks after receiving a booster vaccination.
- Booster vaccination: Those who died after 2 weeks of receiving a booster vaccination on top of receiving a double dose of double-dose vaccines or a dose of single-dose vaccines.

### Statistical Analyses

All data were extracted, cleaned and analysed using R version 4.1.1 (12). Descriptive analysis was conducted to summarise the COVID-19 deaths data. Categorical variables were presented as frequency and percentage. Numerical variables were presented as mean and standard deviation (SD).

Two quasi-Poisson regression analyses were performed to determine the factors associated with COVID-19 deaths given the presence of overdispersion in the data. The first analysis was conducted on the data aggregated by age, gender, comorbidity and vaccination status. The second analysis was conducted on the same aggregated data with adjustment for the percentage of second dose vaccination coverage.

## Results

### Descriptive Statistics

There were 32,240 COVID-19 death cases downloaded from the Ministry of Health Malaysia GitHub website and all the cases were included in the analysis. About 64.9% of the COVID-19 deaths in Malaysia were unvaccinated cases, while the remaining 35.1% were at least partially vaccinated. Generally, most of the COVID-19 deaths in Malaysia were of Malaysian nationality with comorbidities. The percentages of COVID-19 deaths with comorbid conditions were higher in the complete and booster vaccination groups at 90.5% and 93.4%, respectively, while the percentages in the unvaccinated and partial vaccination groups were only at 75.1% and 75.9%, respectively. However, the number of deaths was higher in the unvaccinated and partial vaccination groups compared to the number of deaths in the complete and booster vaccination groups despite a lower percentage of deaths with comorbidities. Table 1 shows the detailed characteristics of all COVID-19 deaths in Malaysia.

### Risk Factors for COVID-19 Mortality

Two quasi-Poisson regression analyses were performed to determine the factors associated with COVID-19 mortality. Table 2 presents the result of the unadjusted quasi-Poisson regression model. However, the estimate could be biased because of the change in the percentage of vaccination coverage over time. Therefore, we included the percentage of second dose vaccination coverage on the date of death in the modelling to account for the possible bias. Table 3 shows the result of the quasi-Poisson regression model, adjusted for the percentage of second dose vaccination coverage.

The adjusted model shows a clearer picture of the risk factor estimates. In the unadjusted model, four estimates were close to 0; the estimates for vaccination status: booster and age: below 5 years old, 6 years old–12 years old and 13 years old–18 years old. Note that these estimates are displayed as 0 in Table 2 owing to the loss of accuracy caused by rounding to two decimal places. After the adjustment, these estimates provided more interpretable incidence rate ratio (IRR) and 95% CIs. Based on the adjusted model, vaccination and lower age showed lower risks of COVID-19 death as indicated by IRR < 1. Being male and having existing comorbidities showed higher risks of COVID-19 death as indicated by IRR > 1.

## Discussion

The objective of this study was to determine the risk factors for COVID-19 mortality. Vaccination status, age groups, gender and presence of comorbidities were found to be the risk factors. The findings showed the benefit of vaccination in reducing the risk of death from COVID-19, especially for those who received the booster dose. We also found that people aged 60 years old and above, being male and having existing comorbidities were associated with a higher risk of death from COVID-19. Those who received complete and partial vaccinations also had higher odds of COVID-19 deaths compared to those who received a booster vaccination.

Unvaccinated male COVID-19 patients with comorbidities aged 60 years old and above were at the highest risk of dying from COVID-19. Studies from Indonesia and the Philippines found a similar pattern, with COVID-19 patients who were male or had a comorbid condition having a higher risk of dying compared to their counterparts (13–15). In terms of age group, the two high-risk groups were those aged 19 years old–59 years old and, 60 years old and over. Thus, the two groups should always be given priority in COVID-19 planning and monitoring. Other studies had explored modifiable risk factors of COVID-19 death, such as vitamin D status, obesity and social deprivation (16–18). However, in our study, vaccination status was the only modifiable risk factor for COVID-19 mortality. The unvaccinated group had a higher number of COVID-19 deaths compared to other vaccination status groups, despite having the lowest percentage of cases with comorbidities (Table 1). The high number of deaths in the unvaccinated group can be attributed to the high number of deaths before the start of the national COVID-19 immunisation programme. However, even after taking into account vaccination coverage with the second dose, our analysis showed a similar picture that proves the benefit of the vaccination.

As of 21 February 2022, about 59.8% of the Malaysian population had received booster doses (19). This study found that the risk of COVID-19 mortality was the lowest among those who received the booster dose, followed by those with complete and partial vaccination statuses. Several studies presented similar findings that a booster dose can protect against COVID-19 infection and severe COVID-19-related outcomes (20–23). In fact, there was evidence that the immunity developed by a complete or primary vaccination wanes after some time (23, 24) and thus a booster dose is recommended especially among the high-risk groups.

Our study had several limitations. Firstly, this study used the latest data published by the Ministry of Health Malaysia. However, the variables included in the analysis were limited to those in the publicly available data. Therefore, we could not examine other modifiable risk factors that could provide additional insights into reducing deaths from COVID-19 in Malaysia. Future studies should explore the effect of modifiable risk factors on COVID-19 mortality in Malaysia. Secondly, the number of COVID-19 deaths among those who received the booster doses was relatively low, which might have affected the regression analysis. However, since the standard error was relatively small compared to the regression coefficient, which in turn resulted in a relatively narrow 95% CI, we considered our results to be reliable despite the small number.

## Conclusion

This study presented the risk factors for COVID-19 mortality, namely vaccination status, age group, gender and the presence of comorbidities. Moreover, it highlighted the benefit of COVID-19 vaccination, particularly by taking the booster dose, to reduce the risk of COVID-19 mortality in Malaysia. The high-risk groups were people over 60 years old, male and with existing comorbidities. In light of these findings, the Malaysian government should step up efforts to ensure everyone over the age of 18 years old in Malaysia receives the booster dose. A targeted awareness campaign is needed to increase awareness of the booster vaccination, especially in the high-risk groups and be better protected from the risk of death from COVID-19.

## Figures and Tables

**Table 1:** Descriptive analysis for all COVID-19 deaths in Malaysia (*N* = 32,240)

Characteristic	Overall *N* = 32,240^a^	Unvaccinated *N* = 20,934^a^	Partial *N* = 6,272^a^	Complete *N* = 4,973^a^	Booster *N* = 61^a^
Dose 1					
AstraZeneca	849 (2.6%)	0 (0.0%)	728 (11.6%)	121 (2.4%)	0 (0.0%)
CanSino	36 (0.1%)	0 (0.0%)	36 (0.6%)	0 (0.0%)	0 (0.0%)
Pending VMS sync	7 (0.0%)	0 (0.0%)	5 (0.1%)	2 (0.0%)	0 (0.0%)
Pfizer	3,689 (11.4%)	0 (0.0%)	2,268 (36.2%)	1,407 (28.3%)	14 (23.0%)
Sinopharm	5 (0.0%)	0 (0.0%)	4 (0.1%)	1 (0.0%)	0 (0.0%)
Sinovac	6,720 (20.8%)	0 (0.0%)	3,231 (51.5%)	3,442 (69.2%)	47 (77.0%)
No vaccine	20,934 (64.9%)	20,934 (100.0%)	0 (0.0%)	0 (0.0%)	0 (0.0%)
Dose 2					
AstraZeneca	147 (0.5%)	0 (0.0%)	26 (0.4%)	121 (2.4%)	0 (0.0%)
Pending VMS sync	5 (0.0%)	0 (0.0%)	1 (0.0%)	4 (0.1%)	0 (0.0%)
Pfizer	1,619 (5.0%)	0 (0.0%)	200 (3.2%)	1,405 (28.3%)	14 (23.0%)
Sinopharm	2 (0.0%)	0 (0.0%)	1 (0.0%)	1 (0.0%)	0 (0.0%)
Sinovac	4,074 (12.6%)	0 (0.0%)	585 (9.3%)	3,442 (69.2%)	47 (77.0%)
No vaccine	26,393 (81.9%)	20,934 (100.0%)	5,459 (87.0%)	0 (0.0%)	0 (0.0%)
Booster					
AstraZeneca	2 (0.0%)	0 (0.0%)	0 (0.0%)	2 (0.0%)	0 (0.0%)
Pfizer	97 (0.3%)	0 (0.0%)	0 (0.0%)	43 (0.9%)	54 (88.5%)
Sinovac	12 (0.0%)	0 (0.0%)	0 (0.0%)	5 (0.1%)	7 (11.5%)
No vaccine	32,129 (99.7%)	20,934 (100.0%)	6,272 (100.0%)	4,923 (99.0%)	0 (0.0%)
Age (years old)^b^	62 (16.2)	61 (17.0)	60 (14.7)	67 (13.4)	67 (13.7)
Gender (Male)	18,518 (57.4%)	11,777 (56.3%)	3,660 (58.4%)	3,040 (61.1%)	41 (67.2%)
BID (Yes)	6,540 (20.3%)	4,630 (22.1%)	1,044 (16.6%)	843 (17.0%)	23 (37.7%)
Malaysian (Yes)	28,385 (88.0%)	17,654 (84.3%)	5,820 (92.8%)	4,856 (97.6%)	55 (90.2%)
Comorbidity (Yes)	25,049 (77.7%)	15,731 (75.1%)	4,762 (75.9%)	4,499 (90.5%)	57 (93.4%)
State					
Johor	3,958 (12.3%)	2,459 (11.7%)	869 (13.9%)	620 (12.5%)	10 (16.4%)
Kedah	2,207 (6.8%)	1,475 (7.0%)	413 (6.6%)	314 (6.3%)	5 (8.2%)
Kelantan	1,292 (4.0%)	845 (4.0%)	163 (2.6%)	284 (5.7%)	0 (0.0%)
Melaka	978 (3.0%)	611 (2.9%)	230 (3.7%)	136 (2.7%)	1 (1.6%)
Negeri Sembilan	1,346 (4.2%)	950 (4.5%)	253 (4.0%)	139 (2.8%)	4 (6.6%)
Pahang	817 (2.5%)	531 (2.5%)	114 (1.8%)	169 (3.4%)	3 (4.9%)
Perak	1,469 (4.6%)	816 (3.9%)	212 (3.4%)	431 (8.7%)	10 (16.4%)
Perlis	139 (0.4%)	76 (0.4%)	22 (0.4%)	41 (0.8%)	0 (0.0%)
Pulau Pinang	1,780 (5.5%)	1,063 (5.1%)	312 (5.0%)	405 (8.1%)	0 (0.0%)
Sabah	2,887 (9.0%)	2,175 (10.4%)	356 (5.7%)	349 (7.0%)	7 (11.5%)
Sarawak	1,622 (5.0%)	819 (3.9%)	117 (1.9%)	682 (13.7%)	4 (6.6%)
Selangor	10,100 (31.3%)	6,755 (32.3%)	2,465 (39.3%)	865 (17.4%)	15 (24.6%)
Terengganu	767 (2.4%)	446 (2.1%)	96 (1.5%)	224 (4.5%)	1 (1.6%)
W.P. Kuala Lumpur	2,705 (8.4%)	1,761 (8.4%)	632 (10.1%)	311 (6.3%)	1 (1.6%)
W.P. Labuan	151 (0.5%)	137 (0.7%)	12 (0.2%)	2 (0.0%)	0 (0.0%)
W.P. Putrajaya	22 (0.1%)	15 (0.1%)	6 (0.1%)	1 (0.0%)	0 (0.0%)

Notes: BID = Brought-in-dead;

a *n* (%);

b mean (SD)

**Table 2:** Risk factors of COVID-19 mortality by quasi-Poisson regression model (*N* = 32,240)

Characteristic	*b*	SE	IRR	95% CI	*z*-stats	*P*-value
Vaccination status						
Unvaccinated		-	-	-	-	
Booster	−5.76	1.07	0.00	0.00, 0.02	−5.39	< 0.001
Complete	−1.43	0.13	0.24	0.18, 0.31	−10.9	< 0.001
Partial	−1.20	0.12	0.30	0.24, 0.38	−10.0	< 0.001
Age (years old)						
Above 60		-	-	-	-	
Below 5	−5.46	1.17	0.00	0.00, 0.02	−4.67	< 0.001
6–12	−6.00	1.39	0.00	0.00, 0.02	−4.32	< 0.001
13–18	−5.52	1.05	0.00	0.00, 0.02	−5.25	< 0.001
19–59	−0.31	0.09	0.73	0.61, 0.88	−3.30	0.002
Gender						
Female		-	-	-	-	
Male	0.30	0.09	1.35	1.12, 1.62	3.20	0.003
Comorbidity						
No		-	-	-	-	
Yes	1.25	0.11	3.48	2.81, 4.35	11.20	< 0.001

Notes: SE = standard error; IRR = incidence rate ratio; CI = confidence interval

**Table 3:** Risk factors of COVID-19 mortality by quasi-Poisson regression model adjusted for the percentage of second dose vaccination coverage (*N* = 32,240)

Characteristic	*b*	SE	IRR	95% CI	*z*-stats	*P*-value
Vaccination status						
Unvaccinated		-	-	-	-	
Booster	−2.05	0.41	0.13	0.05, 0.26	−5.02	< 0.001
Complete	−0.69	0.05	0.50	0.45, 0.56	−12.9	< 0.001
Partial	−0.53	0.05	0.59	0.54, 0.64	−11.6	< 0.001
Age (years old)						
Above 60		-	-	-	-	
Below 5	−2.24	0.45	0.11	0.04, 0.23	−5.03	< 0.001
6–12	−2.41	0.53	0.09	0.03, 0.22	−4.53	< 0.001
13–18	−2.36	0.40	0.09	0.04, 0.19	−5.88	< 0.001
19–59	−0.10	0.04	0.90	0.84, 0.97	−2.91	0.004
Gender						
Female		-	-	-	-	
Male	0.20	0.04	1.23	1.14, 1.32	5.71	< 0.001
Comorbidity						
No		-	-	-	-	
Yes	0.76	0.04	2.13	1.96, 2.32	17.8	< 0.001

Notes: SE = standard error; IRR = incidence rate ratio; CI = confidence interval
